# Expression of Inflammatory Markers in the Muscles of Patients with Idiopathic Inflammatory Myopathy According to the Presence of Interstitial Lung Disease

**DOI:** 10.3390/jcm11113021

**Published:** 2022-05-27

**Authors:** Jung Sun Lee, Byeongzu Ghang, Wonho Choi, Seokchan Hong, Yong-Gil Kim, Chang-Keun Lee, Soo Jeong Nam, Bin Yoo

**Affiliations:** 1 Division of Rheumatology, Department of Internal Medicine, Seoul Veterans Hospital, Seoul 05368, Korea; jungsunlee0820@gmail.com; 2 Division of Rheumatology, Department of Internal Medicine, Asan Medical Center, University of Ulsan College of Medicine, Seoul 05505, Korea; dnjsghaaa@naver.com (W.C.); medivineluke@gmail.com (S.H.); bestmd2000@amc.seoul.kr (Y.-G.K.); cklee@amc.seoul.kr (C.-K.L.); 3 Division of Rheumatology, Department of Internal Medicine, Jeju National University School of Medicine, Jeju National University Hospital, Jeju 63241, Korea; indream81@naver.com; 4 Department of Pathology, Asan Medical Center, University of Ulsan College of Medicine, Seoul 05505, Korea

**Keywords:** myositis, polymyositis, dermatomyositis, interstitial lung disease, HLA-DR antigens

## Abstract

Background: Several studies have identified factors associated with the development of interstitial lung disease (ILD) in patients with idiopathic inflammatory myopathies (IIMs). However, few have assessed the association between ILD and muscle biopsy findings, including inflammatory marker expressions analyzed using immunohistochemistry (IHC). Methods: Muscle biopsies from patients who were newly diagnosed with IIMs between 2000 and 2017 were reviewed. ILD was diagnosed based on chest computed tomography findings at the time of diagnosis of IIMs. IHC staining was performed for CD3, CD4, CD8, CD20, CD68, CD163, MX1, MHC class I, and HLA-DR. The factors associated with the presence of ILD were evaluated by logistic regression analysis. Results: Of the 129 patients with IIM, 49 (38%) had ILD. In the muscle biopsy findings, CD4 expression, MX1 expression on immune cells, and expression of MHC class I and HLA-DR on myofibers were more common in patients with ILD than those without. In the logistic regression analysis, the HLA-DR expression on myofibers was significantly associated with the risk of ILD (OR, 2.39; 95% CI, 1.24–4.90, *p* = 0.012) after adjusting for pathologic findings, clinical features, and autoantibodies. Conclusion: The expression of HLA-DR on myofibers was associated with the presence of ILD in patients with IIM.

## 1. Introduction

Idiopathic inflammatory myopathies (IIMs) are systemic autoimmune disorders characterized by proximal muscle weakness and extra-muscular manifestation [1]. IIM could be classified into dermatomyositis, polymyositis, and inclusion body myositis [2]. Recently, immune-mediated necrotizing myopathy and overlap myositis (including anti-synthetase syndrome) were added to the five main types of IIMs [3]. Anti-synthetase syndrome is characterized by the presence of autoantibodies against aminoacyl-tRNA synthetase and the clinical feature of interstitial lung disease (ILD), myositis, Raynaud’s phenomenon, arthritis, and mechanic’s hand [4]. The lung is the most common extra-muscular target next to the skin, and the frequency of ILD is as high as 20–40% in patients with IIM [5,6]. Because ILD is associated with increased mortality in patients with IIM [7], understanding and predicting the development of ILD in patients with IIM is an important issue. The known factors associated with the risk of ILD are malignancy and autoantibodies including anti-tRNA-synthetase, anti-PM-Scl, anti-Ro-52, and anti-melanoma differentiation-associated protein-5 (MDA5) autoantibodies [8,9,10]. However, the association between the development of ILD and various factors in the muscle, the most important site of involvement in IIMs, is not well-known.

The characteristic histopathologic features of IIMs include the presence of mononuclear inflammatory cell infiltrates in muscle tissue, necrotic muscle fibers, and regenerating muscle fibers [11,12,13]. Recent studies have reported that various inflammatory markers, including major histocompatibility complex (MHC) class, T cell, B cell, macrophage, and type-1 interferon, were associated with IIMs [14,15,16,17,18,19,20,21,22]. Because IIMs with ILD have inflammation in both the muscles and the lungs, the histopathologic features and expression of various inflammatory markers in muscle tissue could be important for understanding the pathogenesis of ILD in patients with IIM.

However, there were no studies that evaluated the association between the presence of ILD in patients with IIM and muscle biopsy findings including inflammatory markers using IHC analysis. Thus, we investigated the factors associated with the presence of ILD, including various inflammatory markers in muscle biopsy specimens using IHC analysis.

## 2. Materials and Methods

### 2.1. Study Population

In this retrospective cohort study, we reviewed the electronic medical records of patients who were newly diagnosed with IIMs and underwent muscle biopsy between January 2000 and April 2017 at the Asan Medical Center, a tertiary referral hospital in Seoul, South Korea. IIMs were diagnosed based on the fulfillment of the 2017 European League Against Rheumatism/American College of Rheumatology classification criteria for adult and juvenile IIM [2]. At the time of diagnosis of IIMs, chest computed tomography (CT) was performed for cancer screening, and the presence of ILD was investigated. Patients under 18 years of age at diagnosis and those diagnosed with inclusion body myositis were excluded.

We gathered information on the following data that were acquired at diagnosis: demographic variables (e.g., age, sex); presence of ILD and dysphagia according to high-resolution CT and videofluoroscopic swallowing study, respectively; and laboratory findings (e.g., myoglobin, creatine kinase, lactate dehydrogenase, autoantibody profiles (i.e., anti-Ro, anti-Jo-1 myositis-specific antibody against the histidyl-tRNA synthetase), C-reactive protein, erythrocyte sedimentation rate).

### 2.2. Histopathologic and Immunohistochemistry Analysis

Muscle biopsy specimen were obtained from the quadriceps or the vasus lateralis muscle. Histopathologic features of muscle biopsy specimens were reviewed by a neuropathologist (SJ Nam), who re-assessed the typical histological findings of inflammatory myositis (e.g., fiber necrosis, fiber size variation (atrophic fibers), internal nuclei, moth-eaten fibers, core-like area and fiber) in each slide. The severity of the histopathologic features was graded from 0 to 3 (0 = none; 1 = mild; 2 = moderate; 3 = severe).

Whole sections of representative formalin-fixed paraffin-embedded tissue blocks of muscle specimens were used for further IHC analysis. IHC analysis was conducted using CD3, CD4, and CD8 as T cell markers, CD20 as a B cell marker, CD68 as a macrophage marker, CD163 as an M2 macrophage marker, MX1 as a systemic inflammatory marker associated with type 1 interferon, HLA-ABC as an MHC class I marker, and HLA-DR as an MHC class II marker.

The following antibodies were used: CD3 (1:100, Rabbit monoclonal, clone POLY, catalog No. A0452, DAKO, Glostrup, Denmark), CD4 (1:16, Rabbit monoclonal, clone SP35, catalog No. 790-4423, Ventana Medical Systems, Tucson, AZ, USA), CD8 (1:400, Mouse monoclonal, clone C8/144B, catalog No. M7103, Cell Marque, Rocklin, CA, USA), CD20 (1:400, Mouse monoclonal, clone L26, catalog No. M0755, DAKO, Glostrup, Denmark), CD68 (1:2000, Mouse monoclonal, clone KP1, catalog No. M0814, DAKO, Glostrup, Denmark), CD163 (1:400, Mouse monoclonal, clone MRQ-26, catalog No. 163M-16, Cell Marque, Rocklin, CA, USA), MX1 (1:500, Rabbit polyclonal, clone N2C2, catalog No. GTX110256, GeneTex, Irvine, CA, USA), MHC Class I (1:10,000, clone EMR8-5, catalog No. ab70328, Abcam, Cambridge, UK), and HLA-DR (1:5000, clone TAL 1B5, catalog No. ab20181, Abcam, Cambridge, UK). IHC staining was performed using a BenchMark XT Autostainer (Ventana Medical Systems, Tucson, AZ, USA). The intensity of IHC markers was graded from 0 to 3 (Grade 0, negative to faint positive and less than 1% extent; Grade 1, weak positive and 2–25% extent; Grade 2, moderate positive and 25–50% extent; and Grade 3, strong positive and more than 50% extent) and Grades 2 and 3 were considered as positive results.

### 2.3. Statistical Analysis

Categorical variables are presented as *n* (%), and continuous variables are presented as median (interquartile range) or mean ± standard deviation. Between-group differences were assessed using Fisher’s exact test or the χ^2^ test for categorical variables and the Mann–Whitney U test or Student’s t-test for continuous variables. Factors significantly associated with ILD were identified using logistic regression, and their odds ratios (ORs) with 95% confidence intervals (CIs) are presented. Variables that were considered clinically important for the treatment outcome and those with a *p* value < 0.2 in univariate analyses were included in the multivariable analysis; in all other analyses, *p* values < 0.05 were considered to denote statistical significance. In multivariable analysis, variables were further selected by backward stepwise regression, and those with *p* values < 0.05 were retained in the final model. All statistical analyses were conducted using R v3.5.1 (The R Foundation for Statistical Computing, Vienna, Austria).

## 3. Results

### 3.1. Comparison of Clinical Features According to the Presence of ILD

Table 1 shows the baseline clinical characteristics, laboratory findings, and pathologic findings according to the presence of ILD. Of the 129 patients with IIM, 49 had ILD (dermatomyositis, 55% (27/66) vs. polymyositis, 45% (22/63), *p* = 0.60). The most common ILD pattern was nonspecific interstitial pneumonia (53%), followed by organizing pneumonia (37%), usual interstitial pneumonia (6%), and unclassified interstitial lung abnormalities (4%). There were no significant differences in the clinical features between the ILD group and the non-ILD group, but the proportions of patients positive for anti-Jo-1 (27% vs. 3%, *p* = 0.001) and anti-Ro (47% vs. 20%, *p* = 0.007) were higher in the ILD group. In addition, CRP was higher in the ILD group as well. The most common histopathologic finding was fiber size variation atrophic fiber, followed by perimyseal lymphocyte, endomyseal lymphocyte infiltration, internal nuclei, and fiber necrosis. The histopathologic features were not significantly different between the ILD group and the non-ILD group.

### 3.2. Immunohistochemistry Staining Features According to the Presence of ILD

**Figure 1:**
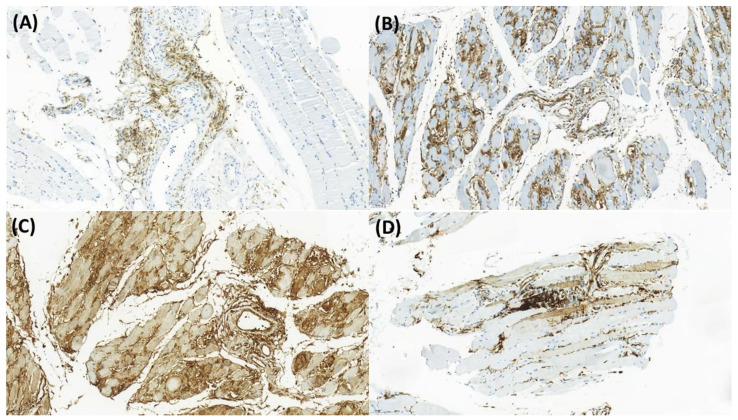
Immunohistochemical staining of CD4, Mx1, MHC class I, and HLA-DR. CD4 expression in the endomysial and perivascular areas (**A**), MX1 expression on immune cells and the capillary of dermatomyositis (**B**), MHC class I expression on immune cells, capillary, and myofibers of dermatomyositis (**C**), and HLA-DR expression on myofibers (**D**) are shown. Magnification, ×20.

Table 2 shows the IHC staining features according to the presence of ILD. The ILD group had higher proportions of patients with CD4 expression (47% vs. 29%, *p* = 0.036), Mx1 expression on immune cells (29% vs. 13%, *p* = 0.023), and expression of MHC class 1 (67% vs. 41%, *p* = 0.004) and HLA-DR on myofibers (37% vs. 11%, *p* = 0.001) than the non-ILD group. Figure 1 shows the representative images of CD4, Mx1, MHC class I, and HLA-DR expression in muscle specimens.

### 3.3. Factors Associated with ILD in Idiopathic Inflammatory Myositis Patients

Logistic regression analysis was performed to evaluate the factors associated with the presence of ILD in patients with IIM. In univariate analysis (Table 3), the titers of anti-Jo-1 and anti-Ro were significantly associated with the presence of ILD. In IHC staining, CD3 or CD4 expression, Mx1 expression on immune cells, and expression of MHC class I or HLA-DR on myofibers were associated with the presence of ILD. In multivariate analysis (Table 4), we created three models that were adjusted for IHC staining (model 1), laboratory finding, histopathologic finding, and IHC staining (model 2), and clinical characteristics, laboratory finding, histopathologic finding, and IHC staining (model 3). HLA-DR expression on myofibers was significantly associated with the presence of ILD in model 1 (OR, 1.97; 95% CI, 1.16–3.49; *p* = 0.015) and model 3 (OR, 2.39; 95% CI, 1.24–4.90; *p* = 0.012). Increases in anti-Jo-1 titer were associated with the presence of ILD in model 2 (OR, 1.02; 95% CI, 1.00–1.05; *p* = 0.058) and model 3 (OR, 1.02; 95% CI, 1.00–1.05; *p* = 0.074), albeit without statistical significance.

## 4. Discussion

By analyzing the expression of inflammatory markers in the muscle specimens of 129 patients with IIM with or without ILD, we found multiple IHC markers (e.g., CD4, MX1 expression on immune cells, expression of MHC class I and HLA-DR on myofibers) that were differentially expressed according to the presence of ILD and significantly associated with ILD in logistic regression analysis. Specifically, regardless of the consideration for histopathologic findings, clinical features, and autoantibodies, HLA-DR expression on myofibers consistently showed a significant association with the presence of ILD.

A study on anti-synthetase myopathy showed that the HLA-DR expression is correlated with CD8+ T cell infiltration, which suggests an involvement of the interferon-gamma pathway in the myofiber HLA-DR expression [23]. Interestingly, a recent study reported that the interferon signature is different according to the type of IIM and that anti-synthetase myopathy showed a prominent type II interferon-gamma signature [24]. In IIM patients with ILD, T cell activation has an important role in lung damage [25], and a common pattern in the T cell receptor gene has been identified in both the lung and muscles [26]. Indeed, in lung biopsy specimens of patients with myositis-associated ILD, CD8+ T cells were increased and diffusely distributed in normal alveoli [27,28]. Furthermore, Jo-1 antigen-reactive CD4+ T cells were found in the bronchoalveolar lavage fluid (BAL) in patients with IIM/anti-synthetase syndrome and CD4+ T cells that produce high levels of interferon-gamma [29]. In addition to these findings, we found that the HLA-DR expression on myofibers is significantly associated with the presence of ILD in patients with IIM. Although interferon-gamma is essential for the repairment of muscles after injury [30], chronic and persistent inflammation could limit muscle repair and lead to muscle atrophy [31]. Furthermore, a previous study reported that the suppression of the interferon-gamma pathway in muscular dystrophy could reduce muscle damage [32]. In IIM, which is a chronic inflammatory condition, interferon-gamma might play a role in the damaging of muscle fibers and the lung. As such, we speculated that HLA-DR expression on myofibers is associated with interferon-gamma-mediated CD8+ or CD4+ T cells that might contribute to the damages in muscle fibers and lungs in IIM patients with ILD. Although the mechanism of the association between myofiber HLA-DR expression and ILD could not be delineated in the present study, our findings provide additional information on the risk and pathogenesis of ILD in IIMs. Further studies are needed to examine the mechanism of the association between myofiber HLA-DR expression and ILD.

There are several studies on the association between the antibodies and ILD in IIMs. Muscle-specific antibodies, including anti-synthetase antibody and MDA-5, were shown to be associated with the presence of ILD and its prognosis [33]. Anti-synthetase antibodies are directed against tRNA-synthetases, whose proteins are not only an essential part of the translation apparatus but also have numerous cytoplasmic, nuclear, and extracellular functions, such as the triggering or silencing of inflammatory/immune responses, participation in lung development, and neuromuscular disorders [34,35]. In addition, histidine, which is incorporated into proteins by histidyl-tRNA-synthetase, is an essential proteinogenic amino acid with an imidazole functional group, which has antioxidant, antisecretory, and anti-inflammatory properties, and suppresses proinflammatory cytokine expression, possibly through the NF-kB pathway [36]. The most common anti-synthetase antibody is the anti-Jo-1 antibody, which specifically recognizes the histidyl-tRNA-synthetase. Therefore, the anti-Jo-1 antibody may have a significant role in autoimmune response. In IIMs, the anti-Jo-1 antibody is most commonly involved in anti-synthetase syndrome but is also found 20–30% in polymyositis [37] and 8.5% in dermatomyositis [38].

Previous studies reported patients with anti-synthetase antibodies with ILD and no myositis [39,40]. While the pathogenesis of anti-synthetase syndrome is suggested to be multifactorial, the lung was suggested as the site of the first lesions. A previous study suggested that the lung might be the primary target of the autoimmune response via a novel antigen including Jo-1 or MDA, and the immune response might be directed against regenerating muscle cells [41]. Indeed, the presence of the anti-Jo-1 antibody in BAL fluid and germinal center-like structures in the lung biopsy in patients with anti-synthetase syndrome suggest that the lung is the primary organ that produces immune responses to histidyl-tRNA synthetase [29]. Furthermore, compared with those from healthy controls, regenerating muscle cells from patients with IIM showed a higher level of histidyl-tRNA synthetase that might play a role as an autoantigen [42]. Interestingly, a recent study showed that the skeletal muscle myoblasts secrete histidyl-tRNA synthetase that inhibits T cells during differentiation into myotubes and that serum histidyl-tRNA synthetase was low in patients with anti-synthetase syndrome [43]. Taken together, the anti-Jo-1 antibody could react with highly expressed histidyl-tRNA synthetase in regenerating muscle, and low serum histidyl-tRNA synthetase could lead to T cell activation and increase the immune response in lungs and regenerating muscles. Accordingly, in the present study, increases in anti-Jo-1 titer were associated with the presence of ILD, albeit without statistical significance. The anti-Jo-1 antibody may have an important role in the development of ILD in IIMs.

The present study has some limitations. First, because the study design was retrospective and performed in a single tertiary referral center, there were missing data regarding autoantibody test results, and other autoantibodies, such as MDA5 and anti-tRNA-synthetase autoantibodies, could not be evaluated. In addition, the histopathologic features of the muscle biopsy and IHC staining grading were reviewed by a single neuropathologist. Second, because we could not evaluate the expression of inflammatory markers and their function in lung biopsy specimens, the mechanism of the association between HLA-DR expression and the presence of ILD could not be explained. However, our study has strength in that we analyzed the muscle specimens from more than 100 cases of IIMs and investigated the expression of various inflammatory markers using IHC staining. Considering the scarcity of studies that utilized IHC staining on muscle specimens of IIM, we believe that the significant associations of various IHC markers (e.g., CD3, CD4, CD20, MX1 expression on immune cells, expression of MHC class I and HLA-DR on myofibers) shown in logistic regression analyses would provide important clues to the pathogenesis of ILD in IIMs, which should be further investigated in targeted studies.

## 5. Conclusions

In patients with IIM, several inflammatory markers were differentially expressed in the muscle according to the presence of ILD. Especially, the expression of HLA-DR on myofibers was consistently associated with ILD, regardless of the consideration of histopathologic findings, clinical features, and autoantibodies. The myofiber expression of HLA-DR could provide additional information on the risk of ILD in patients with IIM and a clue to the pathogenesis of ILD in IIMs.

## Figures and Tables

**Table 1:** Characteristics of idiopathic inflammatory myositis according to the presence of interstitial lung disease.

	ILD (+) (*n* = 49)	ILD (−) (*n* = 80)	*p*
Diagnosis of IIMs			0.60
Dermatomyositis	27 (55%)	39 (49%)	
Polymyositis	22 (45%)	41 (51%)	
ILD pattern			
Nonspecific interstitial pneumonia	26 (53%)		
Organizing pneumonia	18 (37%)		
Usual interstitial pneumonia	3 (6%)		
Unclassified interstitial lung abnormality	2 (4%)		
Clinical characteristics			
Age at diagnosis, years	54 (42–60)	53 (41–64)	0.82
Male sex	18 (37%)	26 (33%)	0.76
Heliotrope rash	10 (20%)	23 (29%)	0.40
Gottron’s sign/papules	14 (29%)	21 (26%)	0.93
V or Shawl sign	7 (14%)	22 (28%)	0.13
Dysphagia	12 (25%)	27 (34%)	0.36
Proximal symmetric weakness	43 (88%)	76 (95%)	0.18
Myopathy on electromyography	35 (80%)	69 (91%)	0.14
Laboratory finding			
Muscle enzyme			
Creatine kinase, U/L	1505 (502–53,290)	2755 (412–7404)	0.48
Myoglobin, ng/mL	559 (196–1714)	752 (223–2219)	0.63
Lactate dehydrogenase, U/L	627 (416–996)	587 (368–1197)	0.88
Autoantibodies			
Anti-Jo (*n* = 108) *	13 (27%)	2 (3%)	0.001
Anti-Ro (*n* = 101) *	21 (47%)	11 (20%)	0.007
ESR, mm/h	51 (36–67)	41 (25–59)	0.15
CRP, mg/dL	1.2 (0.5–3.6)	0.4 (0.1–1.8)	0.003
Histopathologic findings			
Fiber necrosis	23 (47%)	45 (56%)	0.40
Fiber size variation atrophic fibers	46 (94%)	76 (95%)	0.99
Internal nuclei	26 (53%)	45 (56%)	0.86
Moth-eaten fibers	1 (2%)	5 (6%)	0.41
Fiber splitting	8 (16%)	21 (26%)	0.27
Perifascicular atrophy	16 (20%)	7 (14%)	0.56
Endomyseal lymphocyte	53 (66%)	33 (67%)	1.00
Perimyseal lymphocyte	52 (65%)	39 (80%)	0.12

Data are *n* (%) or median (interquartile range). ILD: interstitial lung disease, IIMs: idiopathic inflammatory myopathies, ESR: Erythrocyte sedimentation rate. CRP: C-reactive protein. *: tested number.

**Table 2:** IHC staining results according to the presence of interstitial lung disease ^a^.

	ILD (+) (*n* = 49)	ILD (−) (*n* = 80)	*p*
Inflammatory cell expression			
CD3	25 (51%)	27 (34%)	0.052
CD4	23 (47%)	23 (29%)	0.036
CD8	15 (31%)	14 (18%)	0.08
CD20	12 (25%)	10 (13%)	0.08
CD68	25 (51%)	30 (38%)	0.13
CD163	22 (45%)	32 (40%)	0.72
MX1 expression			
Immune cell	14 (29%)	10 (13%)	0.023
Myofiber	10 (20%)	20 (25%)	0.55
Capillary	34 (69%)	43 (54%)	0.08
Expression of MHC molecules on myofibers			
MHC class 1	33 (67%)	33 (41%)	0.004
HLA-DR	18 (37%)	9 (11%)	0.001

Data are *n* (%). ^a^ Moderate-to-strong intensities of staining were considered as positive results. IHC: immunohistochemistry, ILD: interstitial lung disease, MHC: major Histocompatibility Complex, HLA: human leukocyte antigen.

**Table 3:** Factors associated with the presence of interstitial lung disease in univariate analysis.

	OR	95% CI	*p*
Clinical characteristics			
Age at diagnosis (per 1 year increase)	1.01	0.98–1.03	0.65
Male sex	1.21	0.57–2.54	0.62
Heliotrope rash	0.64	0.26–1.45	0.29
Gottron’s sign/papules	1.12	0.50–2.48	0.77
V or shawl sign	0.44	0.16–1.08	0.09
Dysphagia	0.64	0.28–1.39	0.27
Proximal symmetric weakness	0.38	0.09–1.39	0.15
Myopathy on electromyography	0.39	0.13–1.14	0.09
Myalgia	1.56	0.75–3.22	0.23
Laboratory finding			
Creatine kinase (per 1 U/L increase)	1.00	1.00–1.00	0.29
ESR (per 1 mm/h increase)	1.01	1.00–1.02	0.21
CRP (per 1 mg/dL increase)	0.99	0.89–1.09	0.86
Anti-Ro titer (per 1 U/mL increase)	1.01	1.00–1.01	0.016
Anti-Jo-1 titer (per 1 U/mL increase)	1.03	1.00–1.06	0.023
Histopathologic finding ^a^			
Fiber necrosis	1.09	0.66–1.80	0.73
Fiber size variation atrophic fibers	0.68	0.40–1.15	0.16
Internal nuclei	1.25	0.78–1.99	0.35
Moth-eaten fibers	0.31	0.02–2.02	0.30
Fiber splitting	0.55	0.21–1.32	0.19
Perifascicular atrophy	0.67	0.24–1.71	0.41
Endomyseal lymphocyte	1.10	0.74–1.67	0.63
Perimyseal lymphocyte	1.20	0.92–1.58	0.19
IHC staining ^a^			
Expression of - inflammatory cells			
CD3	1.67	1.03–2.77	0.040
CD4	1.80	1.22–2.72	0.004
CD8	1.43	0.92–2.25	0.11
CD20	1.54	1.03–2.34	0.039
CD68	1.58	0.93–2.72	0.09
CD163	1.42	0.83–2.44	0.20
MX1 expression			
Immune cell	1.89	1.16–3.16	0.012
Myofiber	0.84	0.56–1.22	0.37
Capillary	1.33	0.91–1.97	0.14
Expression of MHC molecules on myofibers			
MHC class 1	1.72	1.11–2.76	0.019
HLA-DR	2.18	1.40–3.53	<0.001

^a^ Relative odds per increase of 1 point in score, range 0–3. ESR: Erythrocyte sedimentation rate. CRP: C-reactive protein, IHC: immunohistochemistry, MHC: major Histocompatibility Complex, HLA: human leukocyte antigen.

**Table 4:** Factors associated with the presence of interstitial lung disease in multivariable analysis.

	Model 1 *			Model 2 ^†^			Model 3 ^‡^		
Factors	OR	95% CI	*p*	OR	95% CI	*p*	OR	95% CI	*p*
V or Shawl sign							0.31	0.08–1.10	0.08
Myopathy on electromyography							0.18	0.02–0.95	0.06
Anti-Jo-1 titer (per 1 U/mL increase)				1.02	1.00–1.05	0.06	1.02	1.00–1.05	0.07
CD4 expression ^a^	1.76	0.96–3.31	0.07						
CD8 expression ^a^	0.56	0.27–1.13	0.11				0.53	0.24–1.10	0.10
MX1 expression on immune cells ^a^	1.51	0.87–2.67	0.15						
HLA-DR expression on myofibers ^a^	1.97	1.16–3.49	0.015	1.62	0.96–2.85	0.08	2.39	1.24–4.90	0.012

* Model 1: adjusted for IHC staining. ^†^ Model 2: adjusted for laboratory finding, histopathologic finding, and IHC staining. ^‡^ Model 3: adjusted for clinical characteristic, laboratory finding, histopathologic finding, and IHC staining. ^a^ Relative odds per increase of 1 point in score, range 0–3.

## Data Availability

The data presented in this study are available upon request from the corresponding author. The data are not publicly available due to patient privacy.
